# Congenital Nephrotic Syndrome With a Novel Presentation in Saudi Arabia

**DOI:** 10.7759/cureus.10222

**Published:** 2020-09-03

**Authors:** Abdulaziz AlHassan, Sajjad M AlKadhem, Fatima Alkhalifah, Jumanah M Almajed, Maryam E Alwabari

**Affiliations:** 1 Pediatrics, Maternity and Children Hospital Al-Ahsa, Al-Ahsa, SAU; 2 Medicine, King Faisal University, Al-Ahsa, SAU

**Keywords:** congenital nephrotic syndrome, saudi arabia, finnish-type congenital nephrotic syndrome, case report, end stage renal disease (esrd)

## Abstract

Congenital nephrotic syndrome (CNS) is a rare and serious entity of renal diseases diagnosed in infants younger than three months. The triad of this syndrome is proteinuria, hypoalbuminemia, and edema. Without renal transplantation, these patients rarely live beyond the age of three years. Infections and sepsis are the most common causes of this condition among children. The majority of patients progress to end-stage renal disease early in life, even with aggressive supportive therapy. In this study, we present a case of a 10-year-old Saudi boy who had been diagnosed with CNS since he was two months old and has improved without renal transplantation.

## Introduction

Congenital nephrotic syndrome (CNS) is defined as heavy proteinuria, hypoalbuminemia, and edema in infants younger than three months of age [1]. Congenital infections, toxoplasmosis, and congenital syphilis are the potential causes of CNS. One of the main types of this syndrome is called the Finnish type, an autosomal recessive disorder. Finnish-type CNS usually results from a monogenic mutation in the NPHS1, which form the glomerular filtration barrier [1-3]. This gene is the most common gene among the Saudi population that causes CNS according to multiple studies performed in different regions of the kingdom [4]. Disruption of normal glomerular filtration results in urinary protein loss, leading to the development of the syndrome's triad [1-3]. Losing proteins such as gamma globulin and anticoagulants cause a decrease in immunity and thrombosis, respectively. Both of these are major complications of the syndrome [5]. Patients with CNS develop end-stage renal disease (ESRD) and rarely live beyond three years of age without renal transplantation [6]. Interestingly, spontaneous resolution of this illness does occur, albeit rarely, as has been reported in many cases [7,8]. This article introduces the reader to the first reported case of a spontaneous partial resolution of Finnish-type CNS in the Middle Eastern countries.

## Case presentation

Our patient was born as a term healthy male from a consanguineous marriage. He had an uneventful prenatal period, with no significant maternal history (four healthy siblings). He was delivered through a spontaneous vaginal delivery. Furthermore, neonatal examinations revealed no dysmorphic features or other abnormalities, and the baby was discharged home with his mother in good condition.

Two months later, he was brought back to the hospital with complaints of generalized body edema, difficulty in breathing, fever, and decreased feeding and activity. Physical examinations showed an edematous infant with reduced activity, irregular breathing, and cyanosis. His vital signs were remarkable for high-grade fever (39°C) and tachycardia (170 bpm). Body fluid samples (blood, urine, and cerebrospinal fluid) were sent for sepsis investigation. The initial laboratory test revealed leukocytosis and elevated inflammatory markers. The patient was started on broad-spectrum antibiotics. Further investigations were conducted, which showed marked hypoalbuminemia, heavy proteinuria, and hypothyroidism; hence, the impression was a CNS. Accordingly, frequent albumin transfusions, captopril, indomethacin, and thyroxine were given to the patient. He was discharged home with indomethacin-captopril in escalating doses according to the clinical response; levothyroxine, alfacalcidol, and iron supplements were also given in addition to aggressive nutritional support.

Outpatient follow-up with the pediatric nephrologist was maintained to receive albumin transfusions followed by furosemide. A genetic study was conducted, which revealed two heterozygous mutations in exon 11 of the NPHS1 gene. Regular monitoring of his biochemistry, blood, and thyroid levels was done. The albumin infusion was given every other day rather than daily at the age of 16 months, and then three days a week, twice weekly, once every two weeks, and once every month at the ages of two, three, four, and five years, respectively.

**Figure 1:**
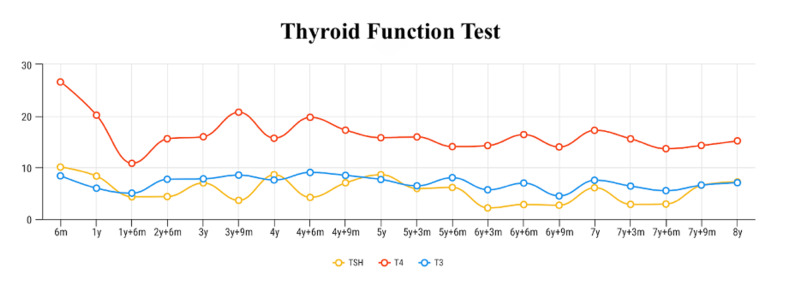
Patient's thyroid function tests throughout the disease course

**Figure 2:**
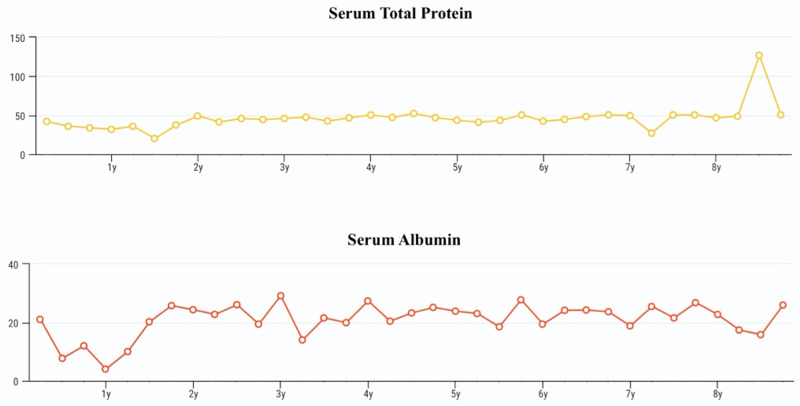
Patient's total serum protein and albumin levels throughout the disease course

**Figure 3:**
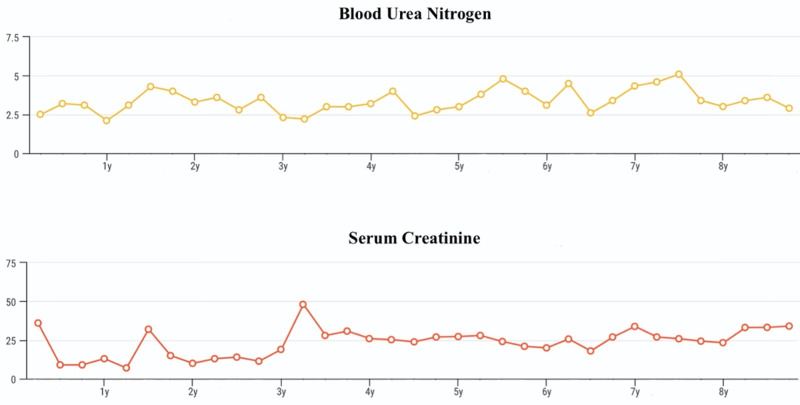
Patient's renal function tests throughout the disease course

At the age of four years, he was diagnosed with bronchial asthma; therefore, captopril was discontinued because of chronic cough. The iron supplement was also suspended as his hemoglobin level normalized. The patient became free from hypothyroidism at the age of eight years, and levothyroxine was discontinued. His thyroid function tests throughout the course of his disease are shown in Figure 1. Gradually, the frequency of the follow-up diverged from weekly to monthly and eventually to once every six weeks. Furthermore, his serum albumin, urinary protein, and renal function tests showed an improvement over time (Figures 2, 3).

Currently, the child is 10 years old and is not on any medication. He has maintained normal renal function and mild hypoalbuminemia and proteinuria (Table 1). He is receiving albumin infusions once every month. He is growing and thriving well with a weight of 30 Kg and a height of 129 cm (25th and 50th percentile, respectively).

**Table 1:** Patient's current lab test. WBC, white blood cell; RBC, red blood cell; HGB, hemoglobin; HCT, hematocrit; MCV, mean corpuscular volume; PLT, platelets

Test	Finding	Reference range
Serum renal function test		
Glucose	101	74-106 mg/dL
Blood urea nitrogen	5.88	1.7-8.3 mmol/L
Serum creatinine	42.76	49-115 umol/L
Calcium	1.99	2.12-2.52 mmol/L
Sodium	140	133-152 mmol/L
Potassium	3.9	3.4-5.2 mmol/L
Magnesium	0.60	0.74-0.99 mmol/L
Phosphate	1.7	0.8-1.5 mmol/L
Iron	12.5	9-31.3 umol/L
Total iron-binding capacity test	38.33	44.75-80.55 umol/L
Serum protein test		
Total protein	51.4	64-82 g/L
Albumin	18.7	34-50 g/L
Urine analysis		
Albumin	+1	-
Pus cells	0-3	<4
Red blood cells	2-4	<5
Glucose	-	-
Acetone	-	-
Cast	-	0-2 hyaline
Crystals	-	No abnormal crystals present
Amorphous salts	-	Few
Urine protein/creatinine ratio	4.6 mg/mg	
Complete blood count		
WBC test	4.67	4.5-13.5 ×10^3^/μL
RBC test	5.45	4-5.2 × 10^6^/μL
HGB	13.4	11.5-15.5 g/dL
HCT	39.3	35-45%
MCV	72.1	75-87 fl
PLT	310	150-450 × 10^3^/μL

## Discussion

Congenital infections, toxins, and idiopathic causes of nephrotic syndrome are usually curable [9]. However, in most cases, CNS is a primary disease of mutation in genes that is necessary for normal glomerular filtration. Among the identified genes, mutations in CNS, NPHS2, and NPHS1 are the most common [9,10]. NPHS1 gene codes for the protein nephrin, which is a major component of the glomerular filtration barrier and abnormalities in this protein and causes the most common cases of CNS in many populations. Our patient’s vital tests excluded the possible secondary causes of CNS, and because he is a product of consanguineous marriage, primary CNS was highly suspected. The genetic study confirmed the hereditary nature of his disease, which revealed two heterozygous mutations in the NPHS1 gene, which might explain the benign course of his illness. There are very few reported cases with similar genetic mutations, and, to our knowledge, this is the first reported case in Saudi Arabia. Lemley reported a case of heterozygous NPHS1 mutation in a patient with mild disease who achieved partial remission with low doses of ACE (angiotensin-converting enzyme) inhibitor and indomethacin [11]. In Saudi Arabia, due to the high prevalence of consanguinity, around 90% of patients diagnosed with CNS have underlying causative mutations with the NPHS1 gene [4].

Disruption of the normal glomerular filtration causes urinary protein loss. Consequently, serum protein and oncotic pressure decrease, thereby causing edema. Among the lost proteins are gamma globulins, which have a role in immune function. Hypogammaglobulinemia put CNS patients at higher risk of infections [9]. The pathophysiology of the disease explains the patient's first presentation with sepsis and generalized edema.

Hypothyroidism, on the other hand, is commonly associated with nephrotic syndrome due to the loss of thyroid-binding globulin in urine as part of the disease pathology [9]. Moreover, the degree of hypothyroidism correlates with the degree of proteinuria and hypoalbuminemia [4]. This might explain our patient's recovery from hypothyroidism.

There are challenges and dilemma in relation to CNS management, especially with the Finnish type. A combination of conservative approach, medical methods, and surgical methods has been advocated. Therefore, dealing with such a condition should be individualized [12,13]. Unlike nephrotic syndrome in older children, CNS has poor outcomes and is usually resistant to steroids and other immunosuppressive treatment [5,10].

Albumin infusion plays a substantial role to compensate for the loss of protein in urine and to prevent hypoalbuminemia complications such as life-threatening edema, malnutrition, and other secondary complications [5]. In our patient, the frequency of the infusion was gradually spaced to almost monthly infusions. Decreasing the need for albumin infusion together with the reduction of hospital visits had positively impacted on the child's psychomotor development and lifestyle, and decreased the risk of the infection [6].

A combination of captopril (ACE inhibitor) and indomethacin (a non-steroidal anti-inflammatory drug) is usually given to the patients as both work as antiproteinuric by decreasing the intraglomerular pressure [10]. A study in New Zealand suggested that genetic background might alter the response to the treatment. They noticed that Maori infants are more likely to respond to the therapy when compared to Caucasian infants [14]. Although it is effective in managing the condition, captopril had to be discontinued in our patient at the age of four because it exacerbated his asthma. Thankfully, the patient's condition did not relapse after the discontinuation of the medication, as seen in one of the reported cases [11].

Patients with CNS usually need renal transplantation or nephrectomy with dialysis and renal replacement therapy to delay mortality due to ESRD [10,14]. A study denoted that around half of CNS patients will require dialysis before the age of two years. Rarely, spontaneous remission of the condition might be seen. However, none of them could identify a predictive factor for the remission [4,7,8].

It is unusual for these patients to be free from medications and not reach ESRD without renal transplantation. It is not known how these patients could survive and improve when compared to other affected children. To the best of our knowledge, our patient is the first case of CNS in Saudi Arabia who survived to this age and became free of medication without the need for renal transplant.

## Conclusions

Sophisticated and individualized management is needed in CNS cases, especially in the Finnish-type. It is anticipated that a child with Finnish-type CNS could develop ESRD. Moreover, it is rare to encounter a spontaneous resolution of a CNS child without a renal transplant. However, our patient achieved that resolution without transplantation while still maintaining normal kidney function and average growth with only minimal supportive therapy. He is the first case to do so in Saudi Arabia. Our case also highlights the importance of genetic testing in predicting the course and outcome of this disease and the success of the conservative approach in the management of such patients.
